# Role of *PCK1* gene on oil tea-induced glucose homeostasis and type 2 diabetes: an animal experiment and a case-control study

**DOI:** 10.1186/s12986-019-0337-8

**Published:** 2019-02-13

**Authors:** Qiantu Hu, Huafeng Chen, Yanli Zuo, Qin He, Xuan He, Steve Simpson, Wei Huang, Hui Yang, Haiying Zhang, Rui Lin

**Affiliations:** 1 Center for Genomic and Personalized Medicine, Guangxi Medical University, 22 Shuangyong Road, Nanning, 530021 Guangxi China; 2 Guangxi Center for Disease Prevention and Control, Nanning, China; 3 General Practice School, Guangxi Medical University, Nanning, China; 4 Melbourne School of Population & Global Health, University of Melbourne, Carlton, Australia; 5 Menzies Institute for Medical Research, University of Tasmania, Hobart, Australia; 6 Public Health School, Guangxi Medical University, 22 Shuangyong Road, Nanning, 530021 Guangxi China

**Keywords:** Fasting blood glucose, Glucose tolerance, Glycolysis/gluconeogenesis pathway, Oil tea, *PCK1*, RT^2^ profiler PCR array, SNP, Type 2 diabetes

## Abstract

**Background:**

Oil tea is a type of traditional tea beverage used for treating various ailments in minority population in Guangxi, China. Our previous study showed oil tea improved glucose and lipid levels in type 2 diabetic mice. Yet, the underling molecular mechanisms are still not understood. This study aimed at assessing the effect of oil tea on glucose homeostasis and elucidating the molecular mechanisms underlying the oil tea-induced antidiabetic effects.

**Methods:**

Twenty seven db/db mice were gavaged with saline, metformin and oil tea for 8 weeks with measurement of biochemical profiles. A real-time^2^ (RT^2^) profiler polymerase chain reaction (PCR) array comprising 84 genes involved in glucose metabolism was measured and validated by quantitative PCR (qPCR). The association between the candidate genes and type 2 diabetes were further analyzed in a case-control study in the Chinese minority population.

**Results:**

Oil tea treatment facilitated glucose homeostasis by decreasing fasting blood glucose and total cholesterol, and improving glucose tolerance. Suppressing phosphoenolpyruvate carboxykinase 1 (*PCK1)* expression was observed in the oil tea treatment group and the expression was significantly correlated with fasting blood glucose levels. Target prediction and functional annotation by WEB-based GEne SeT AnaLysis Toolkit (WebGestalt) revealed that *PCK1* mainly involved in the glycolysis/gluconeogenesis pathway among the top Kyoto Encyclopedia of Genes and Genomes (KEGG) database pathways. Both rs707555 and rs2071023 in *PCK1* were significantly associated with type 2 diabetes in the minority population of Guangxi.

**Conclusion:**

Our findings indicated oil tea improved glucose homeostasis via down-regulation of *PCK1* and *PCK1* may be a genetic marker for the treatment of type 2 diabetes.

**Electronic supplementary material:**

The online version of this article (10.1186/s12986-019-0337-8) contains supplementary material, which is available to authorized users.

## Background

Oil tea is a unique beverage and an important dietary component in the minority areas of Guangxi Province, China. Oil tea is rich in polyphenol, caffeine, 6-gingerol and several minerals [1], and is the most popular traditional beverage used for treating various ailments in Guangxi [2]. Green tea and ginger, as the main ingredients of oil tea, individually have exhibited various biological activities, including anticancer effects, antioxidative effects, and antidiabetic effects [3–5]. Previously, our study showed oil tea treatment improved hyperglycemia, glucose intolerance and hyperlipidemia in db/db mice [1]. While the underlying molecular mechanisms of the beneficial effects of oil tea was unknown.

Phosphoenolpyruvate carboxykinase 1 (*PCK1*) is a key gluconeogenic enzyme in the liver [6]. It is the cytosolic form of phosphoenolpyruvate carboxykinase (PCK or PEPCK) and catalyzes the initial step in hepatic gluconeogenesis. The expression of *PCK1* gene is regulated by insulin, glucocorticoids, glucagon, cyclic adenosine monophosphate (cAMP) and diet, all of which have been considered associated with diabetic conditions. It has been reported that *PCK1* is up-regulated in diabetic rodent models [7], and overexpression in the transgenic mice resulted in insulin resistance [8] and hyperglycemia [9]. Reciprocally, silencing of *PCK1* has been demonstrated to improve glycemia control, insulin sensitivity and dyslipidemia in db/db mice [10]. Furthermore, research has indicated that induced expression of *PCK1* may be a useful indicator for effect assessment in diabetes therapy. Indeed, studies of the effect of Rosmarinic acid, insulin, retinoic acid and phenobarbital showed decreased expression of *PCK1* and improvement of hyperglycemia in diabetic rats [11–13]. Moreover, *PCK1* has been demonstrated to be a candidate genetic marker for diabetes and obesity risk [14], and more recent research implicated this locus in type 2 diabetes (T2D) risk in Chinese [15], South Asian [16] and Finnish populations [17].

In the present study, we investigated the molecular mechanisms of antidiabetic effect of oil tea by examining the expression of *PCK1*, as well as other genes involved in glucose metabolism in db/db mice. We also assessed whether *PCK1* is a genetic marker for T2D risk in the minority population in Guangxi.

## Methods

### Preparation of oil tea

The oil tea was prepared as previously described [1, 18]. In brief, the proportions of green tea, ginger, peanut, cooking oil and salt were 9:10:5:2:1. The manufacturing process was as follows: firstly, soaked green tea leaf was stir-fried and beaten with ginger, peanut and cooking oil in a hot iron pot for 3 min, then hot water and salt added, and the mixture boiled for 1 min and the resulting decoction retained. This process was repeated twice, whereupon the mixed decoction was called oil tea. The oil tea decoction then was concentrated by a rotary vacuum evaporator (IKA Works, Germany) and stored at − 80 °C until use.

### Animal model and experimental design

The experimental procedures were approved by the Animal Ethics Committee of Guangxi Center for Disease Prevention and Control (Approval No. 20160003). Six week-old male diabetic C57BL/KsJ-db/db (db/db) mice were randomly divided assigned to three groups and orally gavaged with oil tea (4 g/kg body weight (BW)/day, *n* = 10), distilled water (0.02 ml/g BW/day, *n* = 7) and metformin (MET, Sigma, USA, the first-line medication for treating T2D, 150 mg/kg BW/day, *n* = 10) for 8 weeks. The oil tea contained 24.6% polyphenols and 0.3% [6]-gingerol, as described previously [1]. Up to five mice per cage were maintained in a temperature-controlled (22 ± 2 °C) mouse facility on a reverse 12-h light/dark cycle and provided water and chow-diet *ad libitum*. The full nutritional composition of oil tea has been described previously [1]. Body weight and food intake were measured each week.

### FBG detection and OGTT test

Fasting blood glucose (FBG) levels were examined every week after the db/db mice overnight fasting. The oral glucose tolerance test (OGTT) was performed at weeks 4 and 8 after the mice were orally gavaged with oil tea or MET, combined with glucose solution (1 g/kg BW). Blood glucose levels were measured in tail blood samples collected at 0, 15, 30, 60, 90 and 120 min after glucose treatment. Blood glucose was measured using a glucometer (OMRON Healthcare Co., Ltd., China).

### Serum lipid measurement

At the end of experiment, mice were killed by cervical dislocation, and blood samples were collected for the measurement of biochemical profiles. Levels of serum triglycerides (TG), total cholesterol (TC), low-density lipoprotein (LDL) and high-density lipoprotein (HDL) were measured using commercial kits (Shanghai Enzyme-linked Biotechnology Co., Ltd., China). Insulin was measured using a commercial Mouse Insulin ELISA kit (ALPCO Diagnostics, USA).

### RT^2^ profiler PCR array analysis

After mice were killed, the fresh livers were collected and snap frozen in liquid nitrogen, and then stored at − 80 °C until analysis. Total RNA was isolated from the liver samples of db/db control group and oil tea group using Qiagen RNeasy® Mini Kit (QIAGEN, Shanghai, China) according to the manufacturer’s instructions. Single-strand cDNA was synthesized from 1 μg of total RNA by reverse transcription reaction using Qiagen RT^2^ First Strand Kit (QIAGEN, Shanghai, China). The cDNA was mixed with Qiagen PCR RT^2^ SYBR Green Master Mix (QIAGEN, Shanghai, China).

To explore the underlying mechanisms of oil tea induced-effects, the expression of 84 genes involved in glucose metabolism including *PCK1* were examined using a Qiagen mouse glucose metabolism RT^2^ profiler PCR array (PAMM-006Z, QIAGEN, Shanghai, China). Relative quantification of mRNA levels was determined by real-time quantitative PCR using a Bio-Rad CFX96 Sequence Detector instrument. The quantitative expression of gene was calculated from the cycle threshold (CT) value of each sample in the linear part of the curve using the relative quantification method (2^−ΔΔCT^) [19]. The samples were analyzed in triplicate and corrected for the selected internal standard which had the smallest standard deviation among the housekeeping genes. Candidate genes were selected from those whose expressions differed greater than 1.25-fold or less than 0.75-fold, or which differed significantly (*p* < 0.05) between the control group and oil tea treatment group.

### Quantitative PCR (qPCR) analysis for the candidate genes

For those candidate genes selected from the RT^2^ profiler PCR array, specific PCR primers were designed for further quantitative real-time PCR analysis (Takara, Dalian, China) as follows.

*ALDOA*: 5^’^- GCTCCTTAGTCCTTTCGCCT - 3^’^ and 5^’^- TCAGTGCTGGGTATGGGTG - 3^’^

*FBP2*: 5^’^- GATCTGTTCATGCTGGACCC - 3^’^ and 5^’^- TACTTGGCATAGCCCTCGTT - 3^’^

*IDH2*: 5^’^- CAGCACTGACTGTCCCCAG - 3^’^ and 5^’^- CACCGTCCATCTCCACTACC - 3^’^

*OGDH*: 5^’^- TGATGATGCTCCGGTAACTG - 3^’^ and 5^’^- AAGTGGTGGTGGGTAAGTGG - 3^’^

*PCK1*: 5^’^- CTGGATGAAGTTTGATGCCC - 3^’^ and 5^’^- TGTCTTCACTGAGGTGCCAG - 3^’^

*PCK2*: 5^’^- GTACTGGGAAGGCATTGACC - 3^’^ and 5^’^- AGTTTGGATGTGCACAGGGT - 3^’^

*PDK2*: 5^’^- ACGTCATTCACTTTCCTCCG - 3^’^ and 5^’^- TGGACATACCAGCTCTGCAC - 3^’^

*PDK4*: 5^’^- AGTGAACACTCCTTCGGTGC - 3^’^ and 5^’^- TGACAGGGCTTTCTGGTCTT - 3^’^

*RBKS*: 5^’^- GTAGTGGTGGGTTCCTGCAT - 3^’^ and 5^’^- CCTTTTCCTCCAAAGCCAA - 3^’^

*UGP2*: 5^’^- CAAGAAAAGGGACCGTCTGT - 3^’^ and 5^’^- GTTATCAGGCAAGCCTCTGG - 3’

cDNA was synthesized with 1 μg RNA using PrimeScript™ RT reagent Kit with gDNA Eraser (Perfect Real Time, Takara, Dalian, China)and mixed with SYBR® Premix Ex Taq™ II (Tli RNaseH Plus, Takara, Dalian, China) according to the manufacturer’s instructions. The quantitative expressions of genes, again, were calculated from the CT value using the 2^−ΔΔCT^ method. Those genes with expressions less than 0.75-fold were selected further for pathway and biological process analysis by using WEB-based GEne SeT AnaLysis Toolkit (WebGestalt, http://www.webgestalt.org/). Target genes were those expression significantly differed between the oil tea group and db/db control group (*p* < 0.05) and were analyzed the correlation with FBG further.

### Selection of the human subjects

Eighty-six T2D patients and 286 healthy subjects were recruited from the nutrition survey conducted in the minority areas in Gongcheng Yao Autonomous County and Binyang County, Guangxi, China from 2014 to 2015. All subjects were conducted with FBG or OGTT test. T2D patients were selected based on the 1999 World Health Organization diagnostic criteria [20]: FBG ≥7.0 mmol/L or OGTT≥11.1 mmol/L or had self-reported diagnosed T2D. Controls were the healthy subjects with normal glucose level: FBG < 6.1 mmol/L and OGTT < 7.8 mmol/L without T2D history. A standard questionnaire was administered by trained staff to obtain information on demographic characteristics, personal and family medical history, etc. This study was approved by the Ethics Committee of Guangxi Center for Disease Prevention and Control (No. GXIRB2016–0014). All subjects provided informed consent.

### Biochemical measurements and genotyping

Overnight fasting blood samples were obtained from cases and controls for biochemical measurement and genotyping. Participants without diagnosed diabetes were given a standard 75-g OGTT with plasma glucose measurement. Plasma glucose of FBG and OGTT were measured using the hexokinase enzymatic method, and serum triglyceride HDL-cholesterol and LDL-cholesterol were assessed using commercially available reagents (Shanghai Kehua Bio-engineering Co., Ltd., China) on an automatic analyzer (Hitachi 7080; Hitachi, Tokyo, Japan). DNA was isolated using the Thermo DNA Blood Kit (ThermoFisher Scientific (China) Co., Ltd., Guangzhou, China). Three polymorphisms (rs707555, rs2071023 and rs2070755) in *PCK1* were selected based on previous research showing them to be significantly associated with T2D [15–17]. SNPs genotyping used the MassARRAY Genotyping System (Sequenom, BGI Tech., Wuhan, China). The primers were designed using MassARRAY Assay Design 3.0 software. The call rate of the genotyping SNPs achieved 99% or higher.

### Statistical analyses

Data were expressed as means ± SD. Comparisons between two groups were performed with two-tailed t-test or Wilcoxon rank-sum test, as appropriate. Datasets that involved more than two groups were assessed by one-way ANOVA followed by Newman–Keuls post-hoc tests. The area under the curve (AUC) of OGTT was calculated by the trapezoid rule [21]. Hardy-Weinberg equilibrium (HWE) was analyzed by using the PLINK [22] software. Logistic regression was used to examine the association between the SNPs and T2D in the case-control analysis. *P*-values less than 0.05 were considered statistically significant. STATA version 12.0 (StataCorp LP, USA) was used for statistical analysis.

## Results

### Oil tea decreases fasting and postprandial glucose

**Fig. 1:**
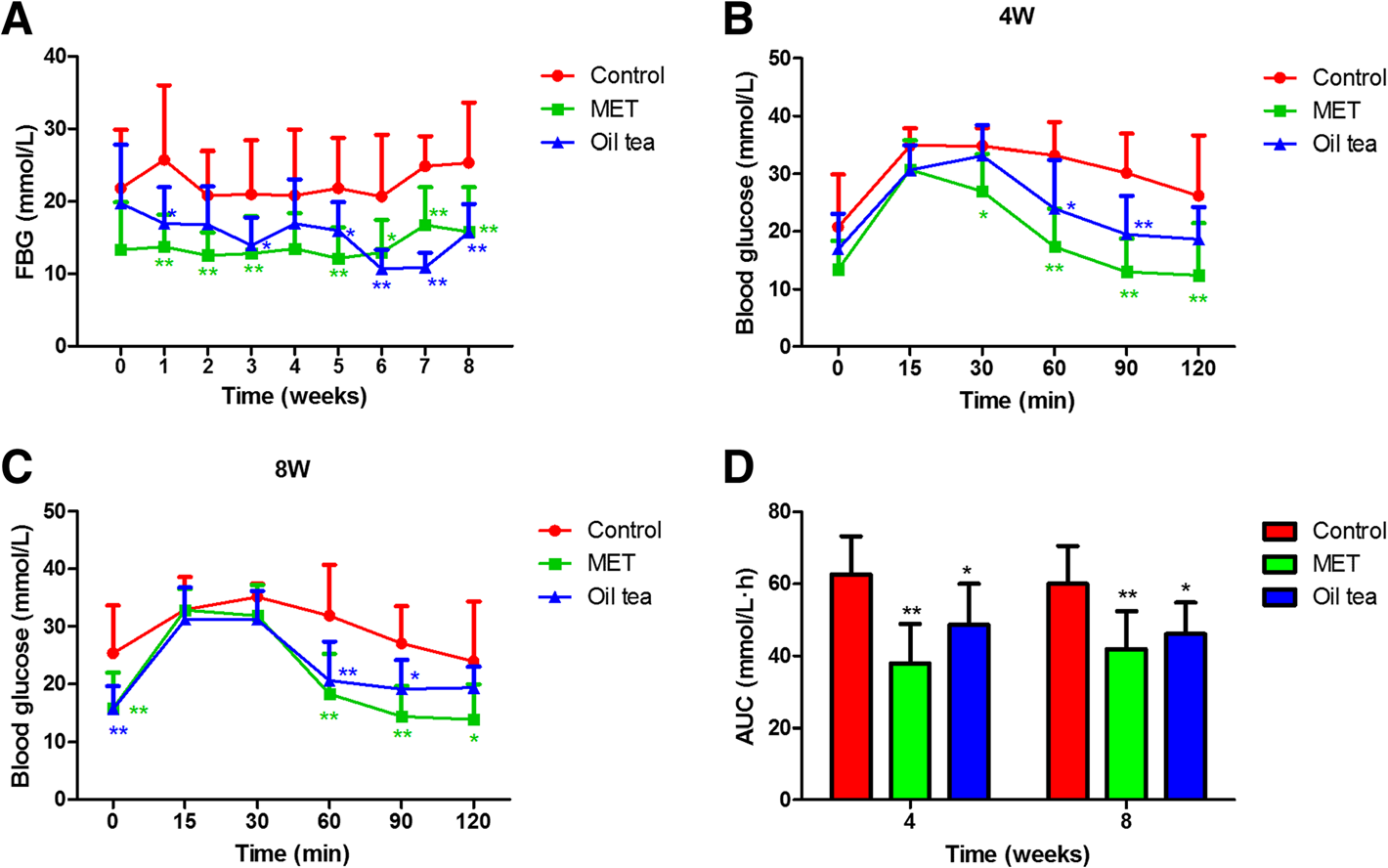
Effect of oil tea on blood glucose in db/db mice. **a** Changes of fasting blood glucose during the 8-week experiment. **b** The glucose levels of the oral glucose tolerance test (OGTT) at week 4. **c** The glucose levels of OGTT at week 8. **d** The AUC levels of OGTT at weeks 4 and 8. FBG, fasting blood glucose; MET, metformin; AUC, area under the curve; control group (*n* = 7); MET group (*n* = 10); oil tea group (*n* = 10). Values are presented as means ± SD. Differences were assessed by one-way ANOVA followed by Newman–Keuls post-hoc tests. *Significantly different from the control group (*p < 0.05, **p < 0.01)

During the 8-week experiment, lower FBG levels were observed in both oil tea and MET treatment group, and a significant decrease was observed from weeks 5 to 8 in comparison with db/db control group (*p* < 0.05). At the end of the experiment, the FBG level of the oil tea group was significantly decreased to 15.68 ± 3.98 mmol/L, compared with the db/db control group of 25.38 ± 8.24 mmol/L (*p* < 0.01). (Fig. 1a).

In the OGTT test, lower glucose levels were observed in both the oil tea and MET treatment groups at weeks 4 and 8, compared to the control group. In particular, significantly lower glucose levels were observed between 60 min and 90 min in the two groups, as well as at 120 min in the MET group (*p* < 0.05). However, no significant differences were observed between oil tea group and control group at 120 min, either at week 4 (*p* = 0.13) or week 8 (*p* = 0.30). (Fig. 1b and c). As for AUC, significantly lower levels were observed in both the oil tea and MET treatment groups at weeks 4 and 8 (*p* < 0.05, Fig. 1d).

### Oil tea lowers biochemical profiles

**Fig. 2:**
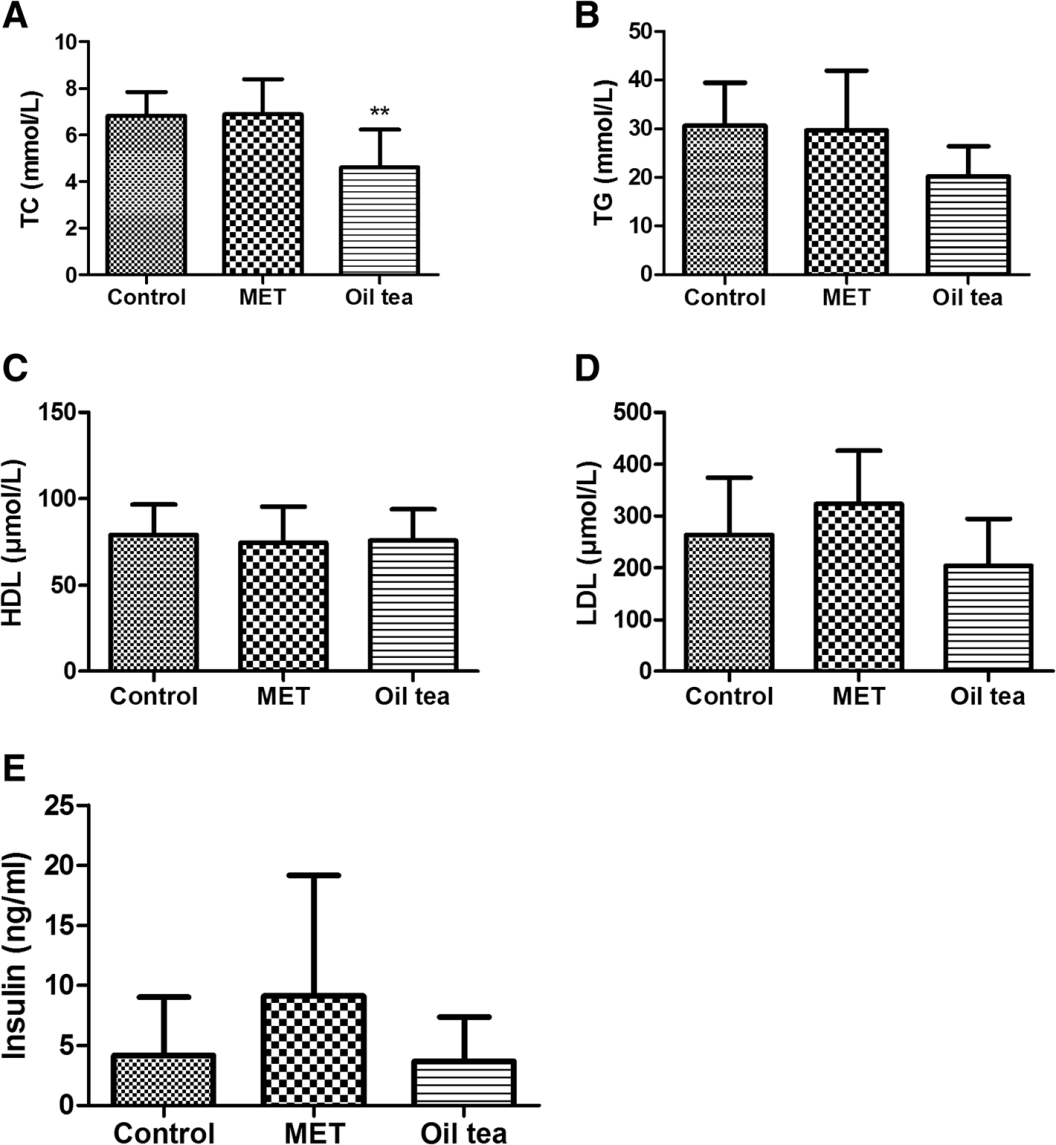
Effect on serum lipid in db/db mice treated with oil tea after 8 weeks. **a** Effect on TC treated with oil tea. **b** Effect on TG treated with oil tea. **c** Effect on LDL treated with oil tea. **d** Effect on HDL treated with oil tea. **e** Effect on insulin treated with oil tea. TC, total cholesterol; TG, triglycerides; LDL, low-density lipoprotein; HDL, high-density lipoprotein; MET, metformin; control group (*n* = 7); MET group (*n* = 10); oil tea group (*n* = 10). Values are presented as means ± SD. Differences were assessed by one-way ANOVA followed by Newman–Keuls post-hoc tests. *Significantly different from the control group (*p < 0.05, **p < 0.01)

As shown in Fig. 2a-e, significantly lower TC levels (*p* < 0.01) and a near-significantly lower TG level (*p* = 0.06) were observed in the oil tea treatment group compared with the control group. Meanwhile, lower levels of LDL (204.28 ± 90.14 vs 263.69 ± 110.40 mmol/L), HDL (75.71 ± 18.16 vs 79.01 ± 17.49 mmol/L) and insulin (3.66 ± 3.68 vs 4.17 ± 4.86 mmol/L) were observed in the oil tea treatment group, although these differences did not reach significance (*p* > 0.05). However, no significant difference was observed between the MET and control groups on any lipid profile (*p* > 0.05).

### No change on food intake and body weight gain with oil tea treatment

**Fig. 3:**
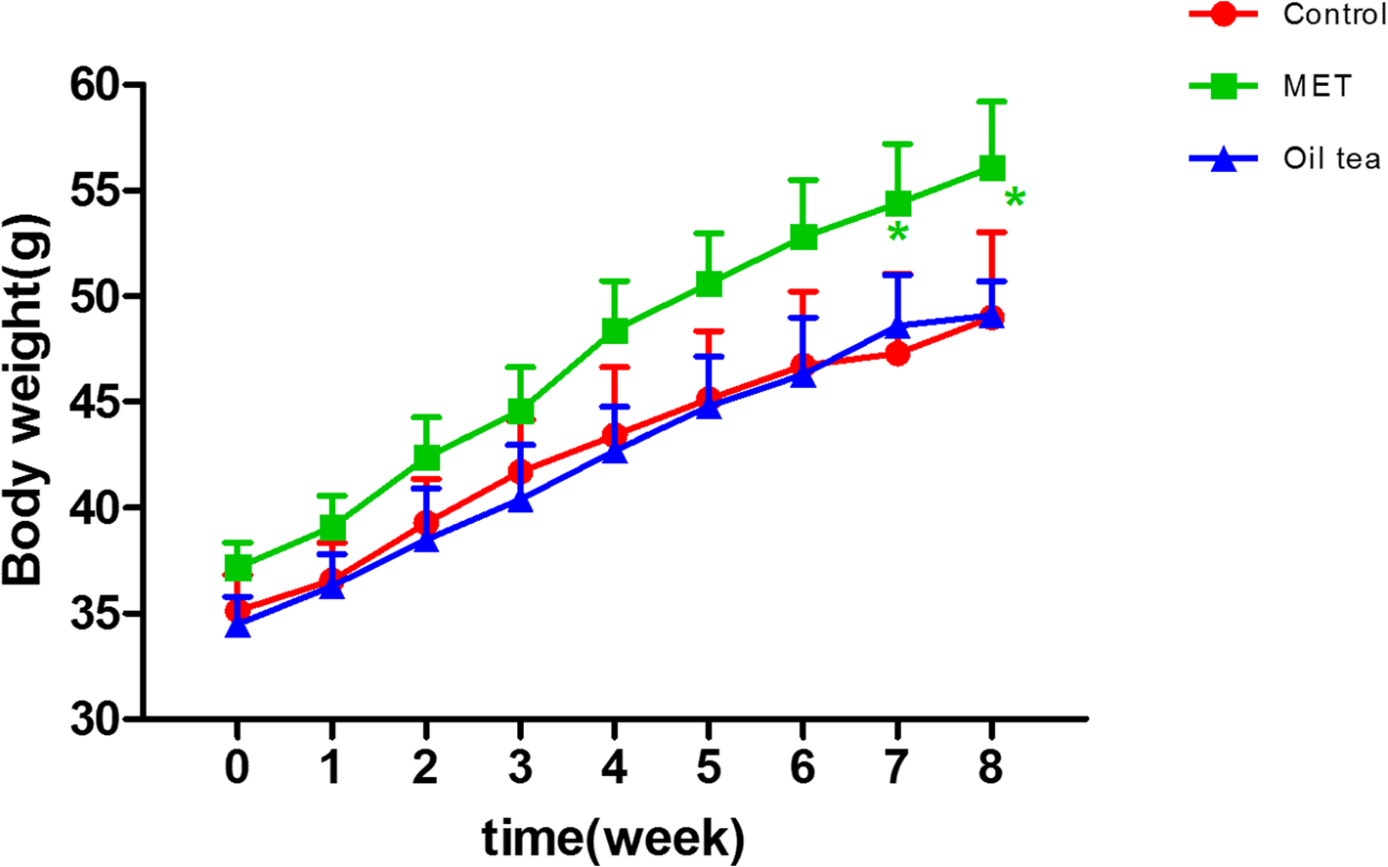
Weekly body weight of mice. MET, metformin; control group (*n* = 7); MET group (*n* = 10); oil tea group (*n* = 10). Values are presented as means ± SD. Differences were assessed by one-way ANOVA followed by Newman–Keuls post-hoc tests. *Significantly different from the control group (*p < 0.05, **p < 0.01)

When the mice were treated with oil tea, overall there were no significant changes along the experiment except significantly lower food intake were observed at weeks 3 (5.21 ± 0.69 vs 6.87 ± 1.00 g, *p* < 0.01) and 5 (4.59 ± 0.83 vs 5.31 ± 1.57 g, *p* < 0.05). When the mice were treated with MET, the food intake was significantly lower at the first week (2.76 ± 2.02 vs 5.32 ± 1.32 g, *p* < 0.05), while no significantly difference was observed at later time points (data not shown). Notably, body weight increased during the experiment in all three groups. No significant differences in weight gain were observed between the oil tea and control groups, but a significantly greater increase was observed in the MET group at weeks 7 and 8 (*p* < 0.05) (Fig. 3).

### Oil tea decreases the expression of those genes involved in glucose metabolism pathway

**Fig. 4:**
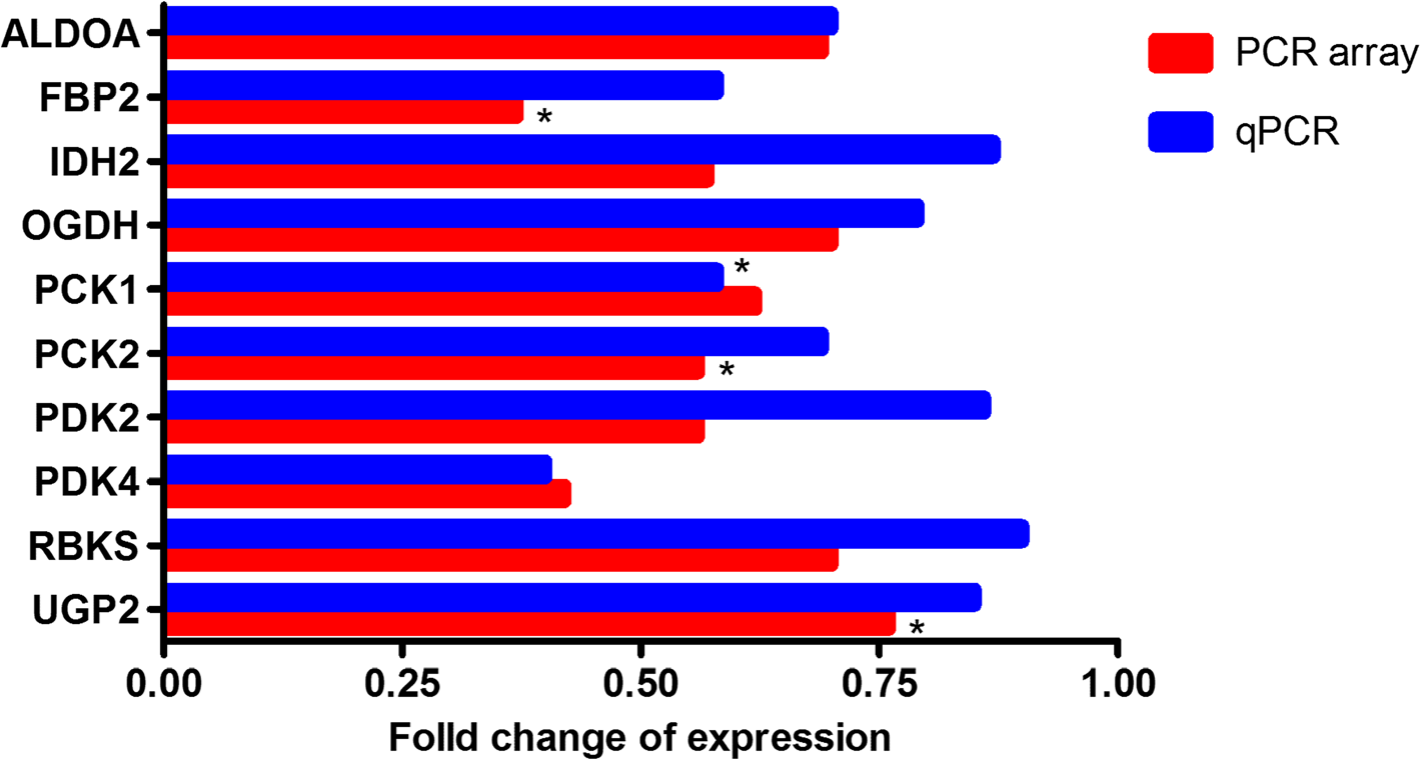
Fold changes of the candidate genes expressions between oil tea and control groups by using RT^2^ profiler PCR array and validated with qPCR. Differences were assessed by t-test or Wilcoxon rank-sum test, as appropriate. *Significantly different from the control group on gene expression (*p < 0.05)

**Fig. 5:**
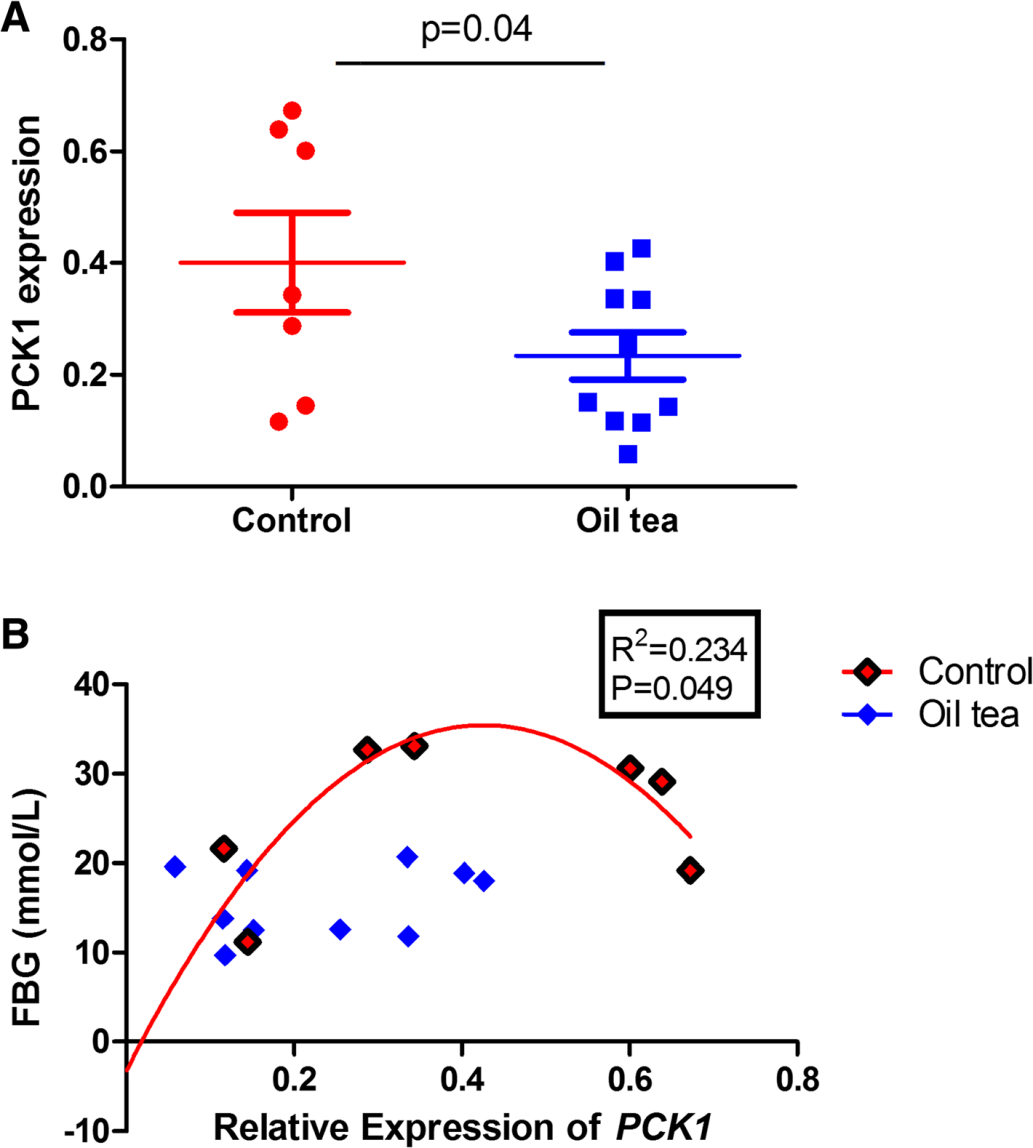
*PCK1* expression responded to oil tea treatment. **a** Decreased hepatic *PCK1* expression in oil tea treatment group. **b** Correlation between *PCK1* expression and fasting glucose levels. FBG, fasting blood glucose; control group (*n* = 7); oil tea group (*n* = 10). Values are presented as means ± SEM. Differences were assessed by t-test. Correlation analysis was performed with Pearson pairwise test

Ten genes involved in the glucose metabolism were detected with different expressions between the oil tea treatment mice and control mice. Out of the 10 genes assessed, 9 genes’ fold change was less than 0.75-fold, and the expression of *UGP2* (fold change = 0.76) was significantly different between oil tea and control groups (*p* = 0.02) (Fig. 4). When the 10 candidate genes were validated by qPCR with the specific primers, we found that all the genes’ expressions were down-regulated, which were consistent with the RT^2^ profiler PCR array results, and the consistent results were observed on the housekeeping genes as well (data not shown). Furthermore, expression of five genes (*PCK1*, *PCK2*, *ALDOA*, *FBP2* and *PDK4*) differed less than 0.75-fold when oil tea treatment group compared with control group **(**Fig. 4). These five genes were further analyzed with WebGestalt into pathway and functional annotation**.** In particular, the expression of *PCK1* was significantly down-regulated after oil tea treatment compared to controls (0.23 ± 0.13 vs. 0.40 ± 0.24, *p* < 0.05, Fig. 5a).

### Association between *PCK1* expression and FBG

We next examined the association between *PCK1* expression and FBG levels. We found that increased *PCK1* expression was significantly correlated with increasing fasting glucose (R^2^ = 0.234, *P* < 0.05, Fig. 5b). However, the expression of other four candidate genes (*ALDOA, FBP2, PCK2* and *PDK4*) were not significantly associated with fasting glucose level (data not shown).

### Prediction and functional annotation by WebGestalt on *PCK1*

**Fig. 6:**
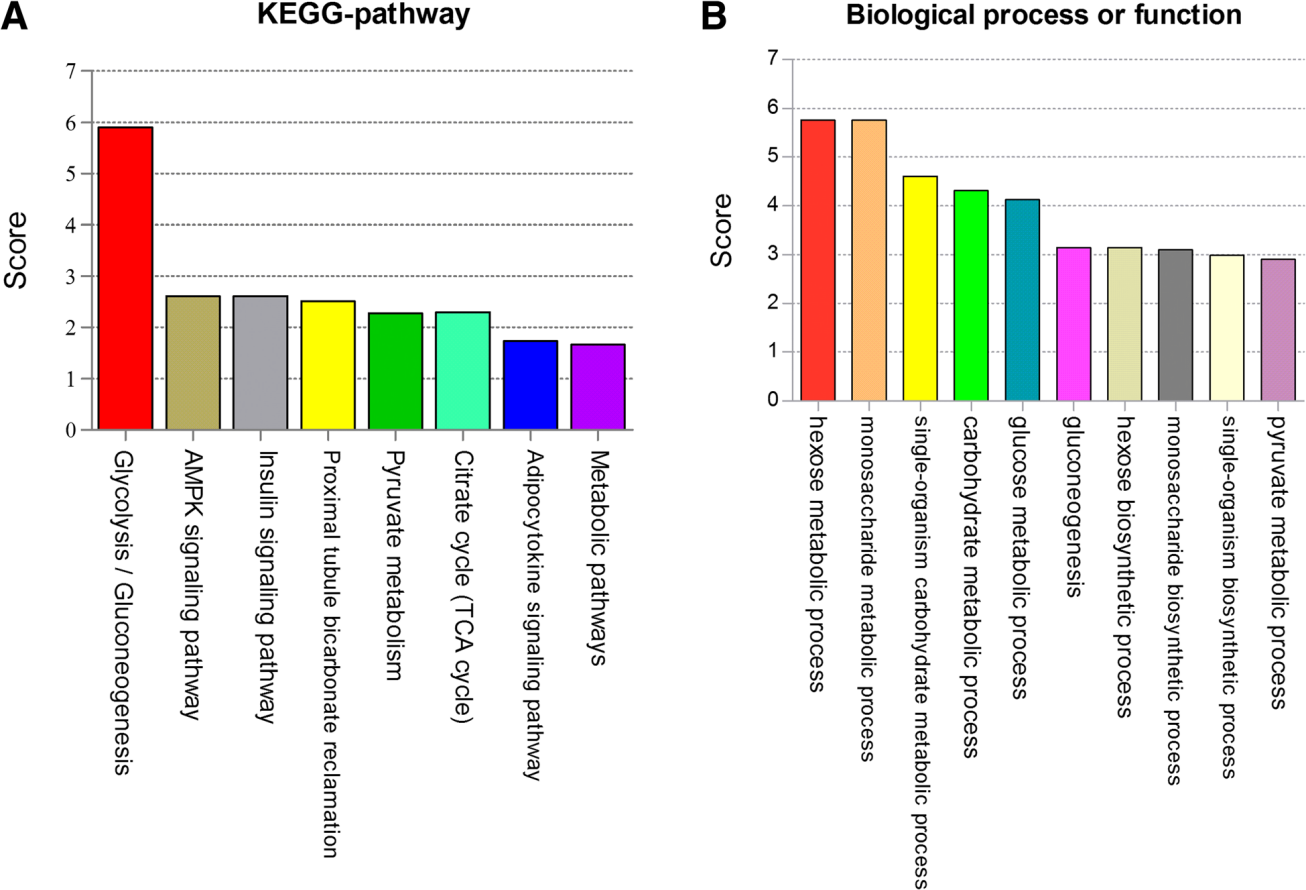
Pathway and functional ranking summary of *PCK*1. **a** Pathway ranking summary of *PCK1*. **b** Functional and biological process ranking summary of *PCK1*. Pathways were annotated according to KEGG, and functional and biological process were annotated by using WebGestalt. Score = −log (false discovery rate, FDR)

By using WebGestalt, the functional annotation results showed that *PCK1* in company with other four candidate genes (*PCK2, ALDOA, FBP2,* and *PDK4*) were mainly involved in glycolysis/gluconeogenesis, AMPK and insulin signaling pathway (Fig. 6a). Analysis on function, biological process showed *PCK1* and the other four candidate genes to be involved in ten types of biological process, including six types of metabolic process (hexose, monosaccharide, glucose, carbohydrate and pyruvate metabolic process), gluconeogenesis and three types of biosynthetic process. (Fig. 6b, Additional file 1: Table S1).

### General characteristics of the human subjects in the case-control analysis

A total of 372 subjects were recruited in the minority areas of Guangxi, China, including 86 T2D cases and 285 healthy controls. The clinical characteristics and biochemical indexes between T2D patients and controls are shown in Table 1. Significant differences were observed in age, sex, BMI, FBG, LDL, TC, TG and anemia between the two groups, all higher among the cases than controls.

**Table 1:** The main phenotypic characteristics of the study participants

	Controls (*n* = 285)	T2D Patients (*n* = 86)	*p*-value
Sex (male/female)	89/196	45/41	< 0.001
Age (years)	53.56 ± 16.33	62.62 ± 11.90	< 0.001
BMI (Kg/m^2^)	22.57 ± 3.60	23.96 ± 3.24	0.003
TG (mmol/L)	1.45 ± 0.84	2.03 ± 1.67	< 0.001
TC (mmol/L)	4.69 ± 1.10	5.26 ± 1.02	< 0.001
FBG (mmol/L)	4.86 ± 0.64	8.57 ± 4.02	< 0.001
HDL-cholesterol (mmol/L)	1.77 ± 0.46	1.72 ± 0.57	0.4025
LDL-cholesterol (mmol/L)	2.27 ± 0.80	2.62 ± 0.80	0.0004
Anemia (*n*, %)	36 (12.9%)	35 (40.7%)	< 0.001
Family history of diabetes (*n*, %)	17 (5.96%)	3 (3.49%)	0.373

*BMI* body mass index, *TG* triglyceride, *TC* total cholesterol, *FBG* fasting blood glucose, *HDL* high density lipoprotein, *LDL* low density lipoprotein

### The association between the *PCK1* SNPs and type 2 diabetes

The three tested SNPs were in Hardy-Weinberg equilibrium with *p* > 0.05. As shown in the Table 2, two SNPs (rs707555 and rs2071023) were significantly associated with T2D, persisting after adjustment for age, sex, BMI, family history of diabetes, TC and TG (P_trend_ = 0.002 and P_trend_ = 0.02, respectively). A clear dose response was observed with the two SNPs as well, such that compared to those non-carrying G allele of rs707555, the subjects carrying one or two of G alleles decreased 62% (OR = 0.38, *p* = 0.002) and 82% (OR = 0.18, *p* = 0.08) risk of T2D, respectively. As for rs2071023, those subjects carrying one or two of G alleles increased 1.94 times (*p* = 0.03) and 2.39 times (*p* = 0.07) risk of T2D, respectively. Meanwhile, a significant negative correlation between rs707555 and FBG level was observed after adjustment for age, sex, BMI, family history of diabetes, TC and TG (P_trend_ = 0.01), as well as a significant positive correlation between rs2071023 and TG level after adjustment for age, sex, BMI and TC (P_trend_ = 0.027). However, no significance was observed between the correlation of 2,071,023 and FBG, rs707555 and TG. No significant associations were observed between rs2070755 and T2D, FBG or TG (Additional file 1: Table S2).

**Table 2:** The association between the rs707555 and rs2071023 in *PCK1* and type 2 diabetes, FBG and TG

Chr/gene	SNP	Allele frequency	Genotype	N	Association with type 2 diabetes		Association with FBG		Association with TG	
					OR (95% CI)	*p-value	Coef. (95% CI)	*p-value	Coef. (95%CI)	& *P*-value
chr20/*PCK1*	rs707555	C (76.62%)	GG	215	ref		ref		ref	
			GC	137	0.38 (0.20, 0.71)	0.002	−0.67 (−1.20, -0.13)	0.015	−0.06 (− 0.28, 0.16)	0.60
		G (23.38%)	CC	18	0.18 (0.05, 1.20)	0.08	−0.79 (−2.03, 0.44)	0.21	−0.39 (− 0.90, 0.11)	0.12
						p-trend = 0.001		p-trend = 0.01		p-trend = 0.20
chr20/*PCK1*	rs2071023	C (65.73%)	CC	153	ref		ref		ref	
			CG	183	1.94 (1.06, 3.58)	0.03	0.51 (−0.03, 1.06)	0.07	0.10(−0.13, 0.32)	0.40
		G (34.27%)	GG	36	2.39 (0.92, 6.22)	0.07	0.30 (−0.61, 1.21)	0.52	0.47 (0.10, 0.85)	0.013
						p-trend = 0.022		p-trend = 0.17		p-trend = 0.027

*p-value adjusted for age, sex, BMI, family-history of diabetes, TG and TC

&*p*-value adjusted for age, sex, BMI and TC

*Chr* chrome, *SNP* single nucleotide polymorphism, *PCK1* Phosphoenolpyruvate carboxykinase 1, *FBG* fasting blood glucose, *BMI* body mass index, *TG* triglyceride, *TC* total cholesterol

## Discussion

In the present study, we have shown that oil tea treatment can impact upon glucose homeostasis in type 2 diabetic mice, as shown by decreased fasting blood glucose and total cholesterol, and improved glucose tolerance. We also identified suppression of hepatic PCK1 in oil tea-treated db/db mice, and *PCK1* expression significantly correlated with the level of fasting blood glucose. *PCK1* was mainly involved in the glycolysis/gluconeogenesis pathway, and two SNPs (rs707555 and rs2071023) in *PCK1* were significantly associated with T2D in the minority population in Guangxi, China.

Oil tea is a type of traditional tea beverage traditionally used in the minority areas in Guangxi Province, China for prevention and treatment of multiple ailments [2]. The main ingredients of oil tea - green tea and ginger, are well-known Chinese Materia Medica [23], having been traditionally used in traditional Chinese medicine and Southeast Asian system medicine [4, 24]. Recent evidence has shown green tea and ginger individually exert antidiabetic effects, including in animal experiments [5, 25–27], cohort studies [28, 29], and randomized controlled trials [3, 30]. As a complex mixture of green tea, ginger and oil, we previously showed that oil tea improved hyperglycemia, glucose intolerance and hyperlipidemia in db/db mice [1]. In the present study, the similar beneficial effects on anti-diabetes of oil tea were observed as well.

To explore the underlying mechanisms of oil tea induced antidiabetic effects, the expression of 84 genes involved in glucose metabolism were examined. Five candidate genes were found with expression changed following oil tea treatment, and they were mainly involved in the glycolysis/gluconeogenesis pathway and participated in different types of metabolism process including glucose metabolic process and gluconeogenesis. It is well established that the rate of gluconeogenesis is modulated by several enzymes, including PCK, fructose-1, 6-biphosphatase, and glucose-6-phosphatase [31–33], in which PCK1 catalyzes the first committed step [34]. In this study, we found that *PCK1* expression was significantly down-regulated after oil tea treatment, and this down-regulation was positive correlated with fasting glucose level in db/db mice. This indicated the observed decreased glucose level may be due to a lower gluconeogenesis rate due to decreased expression of *PCK1*. PCK1 is mainly involved in glycolysis/gluconeogenesis pathways, as well as glucose related-biological process which are involved in glucose homeostasis and type 2 diabetes [35, 36]. These suggest PCK1 may act via glycolysis/gluconeogenesis pathways and thus affect diabetes status during oil tea treatment. Notably, as the first-line medication for treating T2D, MET was designed as the positive control in the animal experiment. It is interesting to find that oil tea showed a cumulative effect on glucose control over days/weeks, while MET affected glucose level much earlier. This indicate MET knocks the gluconeogenesis pathway on the head, while oil tea works in a slower route, for reducing expression of *PCK1* and as it accumulates over days/weeks, glucose levels reduce.

Supporting these results, two SNPs (the missense rs707555 and the promoter rs2071023) in *PCK1* were found to be associated with T2D in minority population in Guangxi, which has a high consumption of oil tea. Previously, research in the Eastern Chinese population showed both of these SNPs were associated with the risk of T2D [15]. Although a different allelic frequency of rs707555 was observed in the minority population of Guangxi, the association between the two SNPs and T2D were consistent with that of in the Eastern Chinese population. Furthermore, we also found that those subjects carrying CG genotype of rs707555 had significantly lower FBG, and people carrying GG genotype of rs2071023 were significantly correlated with higher TG. These findings further supported the association between the two SNPs and T2D since FBG and TG are the key biochemical markers of T2D. Taken together, these results suggest that *PCK1* is a genetic marker of T2D in the Chinese minority population, and thus, interventions which decrease *PCK1* gene expression, such as oil tea consumption, may be effective for T2D prevention or treatment. However, such beneficial effects of oil tea requires investigation in randomized controlled trials.

## Conclusions

Overall, we found that oil tea improved fasting and postprandial glucose and total cholesterol. We have identified a potential molecular mechanism underlying oil tea induced-antidiabetic effects through which expression of *PCK1* was reduced. Our findings suggest *PCK1* plays a key role in the beneficial effects of oil tea, and *PCK1* may be a potential genetic marker for the treatment of type 2 diabetes. Randomized controlled trials to substantiate the clinical efficacy of oil tea in T2D are required.

## Additional file

Additional file 1: **Table S1.** The enriched terms of differential expression genes by using WebGestalt. **Table S2.** The association between the rs20707555 in *PCK1* and type 2 diabetes, FBG and TG. (DOC 68 kb)

## Abbreviations

AUC: area under the curve
BW: body weight
CT: cycle threshold
FBG: fasting blood glucose
FDR: false discovery rate
HWE: Hardy-Weinberg equilibrium
KEGG: Kyoto Encyclopedia of Genes and Genomes
MET: metformin
OGTT: oral glucose tolerance test
*PCK1*: Phosphoenolpyruvate carboxykinase 1
PCR: polymerase chain reaction
qPCR: quantitative PCR
RT: real-time
SNP: single nucleotide polymorphism
T2D: type 2 diabetes
TC: total cholesterol
TG: triglycerides
WebGestalt: WEB-based GEne SeT AnaLysis Toolkit
