# Supplementary material for: Role of PCK1 gene on oil tea-induced glucose homeostasis and type 2 diabetes: an animal experiment and a case-control study
Source: Nutr Metab (Lond). 2019 Feb 13;16:12. doi: 10.1186/s12986-019-0337-8 (PMC6373102; doi:10.1186/s12986-019-0337-8)
Supplement: Supplementary file 1 — Table S1. The enriched terms of differential expression genes by using WebGestalt. Table S2. The association between the rs20707555 in PCK1 and type 2 diabetes, FBG and TG. (DOC 68 kb) [file 12986_2019_337_MOESM1_ESM.doc]

**Additional file 1: Table S1 The enriched terms of differential expression genes by using WebGestalt**

|  | **Term** | **Gene count** | **P-value** | **Genes** | **FDR** |
| --- | --- | --- | --- | --- | --- |
| **Pathway** |  |  |  |  |  |
| KEGG_pathway | Glycolysis / Gluconeogenesis | 4 | 4.2e-09 | ALDOA, FBP2, PCK2, PCK1 | 1.25e-06 |
| KEGG_pathway | AMPK signaling pathway | 3 | 1.94e-05 | FBP2, PCK2, PCK1 | 2.43e-03 |
| KEGG_pathway | Insulin signaling pathway | 3 | 2.44e-05 | FBP2, PCK2, PCK1 | 2.43e-03 |
| KEGG_pathway | Proximal tubule bicarbonate reclamation | 2 | 4.15e-05 | PCK2, PCK1 | 3.09e-03 |
| KEGG_pathway | Citrate cycle (TCA cycle) | 2 | 1.18e-04 | PCK2, PCK1 | 5.01e-03 |
| KEGG_pathway | Pyruvate metabolism | 2 | 1.43e-04 | PCK2, PCK1 | 5.31e-03 |
| KEGG_pathway | Adipocytokine signaling pathway | 2 | 5.58e-04 | PCK2, PCK1 | 1.85e-02 |
| KEGG_pathway | Metabolic pathways | 4 | 7.78e-04 | ALDOA, FBP2, PCK2, PCK1 | 2.18e-02 |
| **Function, biological process** | | | | | |
| [GO:0019318](http://amigo.geneontology.org/amigo/term/GO:0019318) | hexose metabolic process | 5 | 2.35E-10 | ALDOA, FBP2, PCK2, PCK1, PDK4 | 1.74E-06 |
| [GO:0005996](http://amigo.geneontology.org/amigo/term/GO:0005996) | [monosaccharide metabolic process](http://amigo.geneontology.org/amigo/term/GO:0005996) | 5 | 4.37E-10 | ALDOA, FBP2, PCK2, PCK1, PDK4 | 1.74E-06 |
| GO:0044723 | [single-organism carbohydrate metabolic process](http://amigo.geneontology.org/amigo/term/GO:0044723) | 5 | 9.33E-09 | ALDOA, FBP2, PCK2, PCK1, PDK4 | 2.48E-05 |
| [GO:0005975](http://amigo.geneontology.org/amigo/term/GO:0005996) | [carbohydrate metabolic process](http://amigo.geneontology.org/amigo/term/GO:0005975) | 5 | 2.45E-08 | ALDOA, FBP2, PCK2, PCK1, PDK4 | 4.89E-05 |
| GO:0006006 | [glucose metabolic process](http://amigo.geneontology.org/amigo/term/GO:0006006) | 4 | 4.72E-08 | FBP2, PCK2, PCK1, PDK4 | 7.53E-05 |
| GO:0006094 | [gluconeogenesis](http://amigo.geneontology.org/amigo/term/GO:0006094) | 3 | 5.37E-07 | FBP2, PCK2, PCK1 | 7.12E-04 |
| GO:0019319 | [hexose biosynthetic process](http://amigo.geneontology.org/amigo/term/GO:0019319) | 3 | 6.25E-07 | FBP2, PCK2, PCK1 | 7.12E-04 |
| GO:0046364 | [monosaccharide biosynthetic process](http://amigo.geneontology.org/amigo/term/GO:0046364) | 3 | 7.91E-07 | FBP2, PCK2, PCK1 | 7.89E-04 |
| GO:0044711 | [single-organism biosynthetic process](http://amigo.geneontology.org/amigo/term/GO:0044711) | 5 | 1.18E-06 | ALDOA, FBP2, PCK2, PCK1, PDK4 | 1.04E-03 |
| GO:0006090 | [pyruvate metabolic process](http://amigo.geneontology.org/amigo/term/GO:0006090) | 3 | 1.57E-06 | ALDOA, PCK1, PDK4 | 1.25E-03 |
| FDR: false discovery rate; WebGestalt: WEB-based GEne SeT AnaLysis Toolkit (<http://www.webgestalt.org/>); KEGG: Kyoto Encyclopedia of Genes and Genomes | | | | | |

**Table S2 The association between the rs20707555 in *PCK1* and type 2 diabetes, FBG and TG**

| Chr/gene | SNP | Allele frequency | Genotype | N | Association with type 2 diabetes |  | Association with FBG |  | Association with TG |  |  | |
| --- | --- | --- | --- | --- | --- | --- | --- | --- | --- | --- | --- | --- |
|  |  |  |  |  | OR (95% CI) | *p-value | Coef. (95%CI) | *p-value | Coef.(95%CI) | &p-value |  | |
| chr20/*PCK1* | rs2707555 | C (67.20%) | CC | 163 | ref |  | ref |  | ref | ref |  | |
|  |  |  | CG | 174 | 1.17（0.66,2.06） | 0.59 | 0.15（-0.38,0.69） | 0.57 | -0.06(-0.28,0.16) | 0.58 |  | |
|  |  | G (32.80%) | GG | 35 | 0.98（0.34,2.89） | 0.98 | -0.04（-0.96,0.88） | 0.93 | 0.16(-0.22,0.54) | 0.42 |  | |
|  |  |  |  |  |  | p-trend=0.76 |  | p-trend=0.81 |  | p-trend=0.78 |  | |
| * P-value adjusted for age, sex, BMI, family-history of diabetes, TC and TG. | | | | | | | | | | | |  |
| &P-value adjusted with age, sex, BMI and TC. | | | | | | | | | | | |  |
| Chr=chrome; SNP=single nucleotide polymorphism; PCK1=Phosphoenolpyruvate carboxykinase 1; FBG=fasting blood glucose; BMI=body mass index; TG=triglyceride; TC=total cholesterol. | | | | | | | | | | | |  |
